# Novel Strategy to Evaluate Infectious Salmon Anemia Virus Variants by High Resolution Melting

**DOI:** 10.1371/journal.pone.0037265

**Published:** 2012-06-12

**Authors:** Dagoberto Sepúlveda, Constanza Cárdenas, Marisela Carmona, Sergio H. Marshall

**Affiliations:** 1 Laboratorio de Patógenos Acuícolas, Núcleo Biotecnología Curauma, Pontificia Universidad Católica de Valparaíso, Campus Curauma, Valparaíso, Chile; 2 Laboratorio de Genética e Inmunología Molecular, Instituto de Biología, Facultad de Ciencias, Pontificia Universidad Católica de Valparaíso, Campus Curauma, Valparaíso, Chile; 3 Núcleo Biotecnología Curauma, Pontificia Universidad Católica de Valparaíso, Valparaíso, Chile; Auburn University, United States of America

## Abstract

Genetic variability is a key problem in the prevention and therapy of RNA-based virus infections. Infectious Salmon Anemia virus (ISAv) is an RNA virus which aggressively attacks salmon producing farms worldwide and in particular in Chile. Just as with most of the *Orthomyxovirus*, ISAv displays high variability in its genome which is reflected by a wider infection potential, thus hampering management and prevention of the disease. Although a number of widely validated detection procedures exist, in this case there is a need of a more complex approach to the characterization of virus variability. We have adapted a procedure of High Resolution Melting (HRM) as a fine-tuning technique to fully differentiate viral variants detected in Chile and projected to other infective variants reported elsewhere. Out of the eight viral coding segments, the technique was adapted using natural Chilean variants for two of them, namely segments 5 and 6, recognized as virulence-associated factors. Our work demonstrates the versatility of the technique as well as its superior resolution capacity compared with standard techniques currently in use as key diagnostic tools.

## Introduction

Infectious Salmon Anemia Virus (ISAv) is a very aggressive RNA virus which particularly affects salmon fish production in confined conditions. It was first described in Norway [1], and then in Canada [2]–[4], USA [5], Scotland [6], Faroe Islands [7] and finally in Chile [8], [9]. The etiological agent belongs to the family of the *Orthomyxoviridae*, the same as that of animal and human influenza, and to date it is the only reported member of the genus *Isavirus* [10], [11]. The virus is exclusively restricted to fish and does not represent a menace to other animal species. The ISAv genome consists of eight single-stranded RNA segments with negative polarity coding for at least 10 viral-specific proteins [12], [13] and, as with most *orthomyxoviruses*, it displays high variability, representing potential differences in virulence, thus challenging salmon rearing sustainability. Pathogenicity of this family of viruses, using influenza as a model [14], seems to be determined by the interaction of multiple viral-specific coded genes [14], taking as examples the case for the hemagglutinin [15], the NS1 [16], the PB1 [17] and the Neuroaminidase proteins [18]. In contrast, the only confirmed pathogenically associated protein in the ISA virus is hemagglutinin-esterase (HE), which is encoded in viral segment 6 displaying both receptor binding and receptor destroying activity [19], [20]. In addition, at its C-terminal end HE contains a highly polymorphic region (HPR) 35 amino acids in length, close to the transmembrane domain, the target for deletions which originate all reported virulent variants [21]–[24]. To date over 30 different HPR variants have been described worldwide and intriguingly, the variant which contains the complete amino acidic sequence in the HPR region, named HPR0, is the only variant reported which does not produce the disease *in vivo* and it has not yet been possible to grow *in vitro* [25]–[28]. In fact, all virulent ISAv variants recovered from sick fish as infective units contain deletions in the HPR region and can be grown in susceptible cell lines. Recently, the viral fusion protein coded in segment 5 was also associated with virulence [29], [30]. This protein is known to be involved in viral-host cell endosomal membrane fusion. The protein is synthesized as a precursor F_0_, which is proteolytically cleaved to F1 and F2, and held together by disulfide bridges [31]. One of the distinctive features of the coding sequence for the protein is that it is prone to site-directed transpositional events and at least four different inserts have been characterized near the cleavage site of the protein with the distinctive feature that all of them involve endogenous viral sequences. Of these, insert IN1 derives from viral segment 3, inserts IN2 and IN3 from viral segment 5 while insert IN4 is derived from viral segment 2 and is the only one specifically associated with Chilean isolates [25], [32]. Although at present there is no clear evidence confirming that these variations are directly associated with pathogenicity, non-infectious HPR0 has been reported to contain a key amino acid (Q at position 266 in the mature F protein, numbering according sequence EU851044) while virulent forms of the virus contain amino acid L at this position. This suggests that the change at this position could be considered as a prerequisite for virulence development. This evidence also suggests that the presence of Q plus an insertion might favor the accession to increased virulence [29].

Due to the multiple factors putatively associated in defining virulence of ISAv *in vivo,* molecular diagnosis is not a simple task. Of a number of available techniques, detection is primarily carried out via qRT-PCR [33], which in spite of its unquestionable advantages, only provides information on the presence or absence of viral sequences in fish from the field. Notwithstanding, in order to define which variant you are dealing with, an array of distinguishing features need to be known in order to properly characterize each isolate. Thus, it appears to be necessary to design and develop new strategies to properly and accurately evaluate variant infectivity as well as to provide relevant information to improve regulations to sustain confined fish management.

High Resolution Melting (HRM) is a PCR-based procedure which works via the inclusion of saturating dyes during the amplification process [34], [35]. This then allows accurate measurement of a fluorescent melting curve as a result of the transition from double-stranded DNA (dsDNA) to single-stranded DNA (ssDNA) on the basis of temperature increase. Under these conditions small variations in the melting patterns of the resulting amplicons are a reflection of tiny variations in the coding sequences. HRM has been used widely to type Single Nucleotide Polymorphism (SNP) [36], differentiate gene mutations [37], [38], detect tandem repeats [39], and to detect and identify microbes [40]–[42]. It has also been applied to the characterization and genotyping analysis of certain RNA viruses such as noro [43], bursal disease [44] and influenza [45].

In this report we have successfully adapted HRM to differentiate between ISAv segments 5 and 6 as putative virulence-associated molecular markers, thus constituting a novel complementary procedure to full differentiation of ISAv variants in Chile.

## Results and Discussion

**Figure 1:**
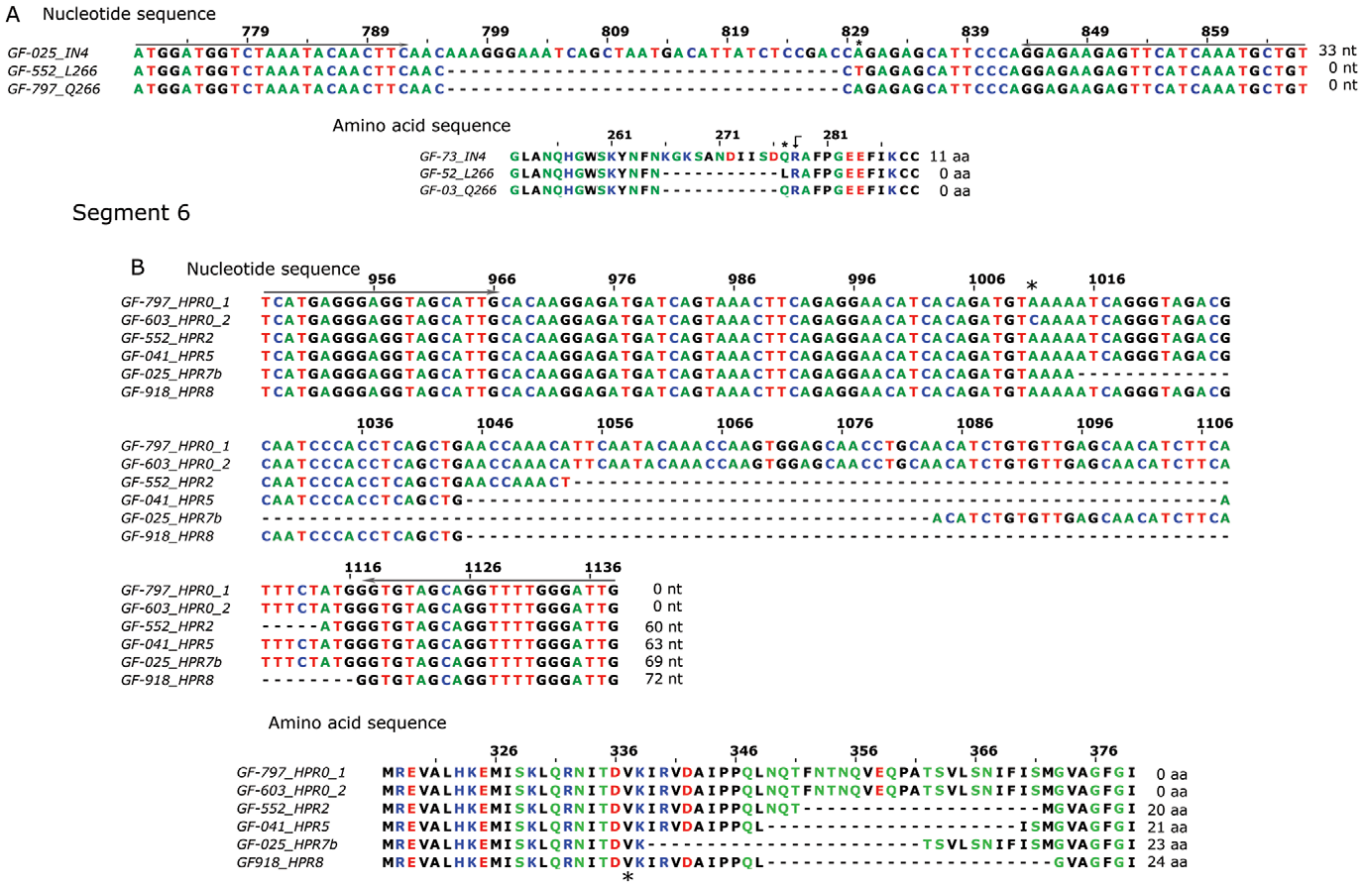
Schematic of nucleotide and amino acid sequences of analyzed viral regions of segments 5 and 6. The number of nucleotides and amino acids indicated at the end of the sequences correspond to the number inserted in segment 5 and deleted from segment 6. The grey arrow above the nucleotide sequence in each segment shows the primer locations. The asterisk marks the site of nucleotide change and the black dot indicates the cleavage site in the fusion protein (segment 5) [46], [47].

In this study we have used HRM as a novel procedure for analysis and differentiation of ISAv variants targeting segments 5 and 6 of the virus and focusing on key regions in both segments which have been associated with virulence [25], [29]. Table 1 shows the characteristics of the resulting amplicons from different samples analyzed by HRM. Figure 1 shows reported target nucleotide and amino acidic sequences involved in the analysis for both segments.

**Table 1:** Characteristics of the amplicons submitted to HRM analysis.

Segment	Primer Sequences 5′–3′	Samples	Variant	GenBank Accession Number	Amplicon Size
**5**	**GIM seg5 5F** ATGGATGGTCTAAATACAACTTC	GF-025	Insert IN4	EU130923	98
	**GIM seg5 1R** ACAGCATTTGATGAACTCTTCTC	GF-797 GF-603	No insert Q_266_	EU851044	65
		GF-552 GF-918	No insert L_266_	GU830907	65
**6**	**GIM seg6 3F** TCATGAGGGAGGTAGCATTG	GF-797 GF-603*	HPR0	EU118820	192
	**GIM seg6 5R** CAATCCCAAAACCTGCTACAC	GF-552	HPR2	AF364889	132
		GF-041	HPR5	EU625673	129
		GF-025	HPR7b	FJ594319	123
		GF-918	HPR8	AY973192	120

* The sequence is identical to EU118820 except for the single nucleotide change of A to C (Figure 1B *).

### Primer Design

Several sets of primers were designed on the basis of conserving flanking sequences using those available in the GenBank database as reference [48]. For segment 5, close to the insertion/cleavage site; and for segment 6, including the HPR region.

Five primer pairs were tested for segment 5 and six primer pairs for segment 6. The correct primer choice is critical to increasing the resolution of the variants since small variations in their sequences can dramatically affect results. Thus, primer selection is a pivotal requirement for accuracy and selection was made according to the keen specificity (single band in agarose gels) and based on their ability to resolve the different variants available (Figure 1). Table 1 shows the selected primer sets based upon their specificity, segregation capacity and accurate reproducibility in the HRM analyses.

### PCR Optimization Conditions

Due to the heterogeneity of the analyzed samples (origin, infection level, processing batch) and taking into consideration that HRM is influenced by factors such as salt concentration and DNA content [49], it was compulsory to perform a primary PCR. This was done to standardize initial conditions of amplification thus avoiding bias in the final analysis. All samples were submitted to the same procedure prior to HRM analysis. In order to project HRM analysis to field studies, samples with known sequences should be included in this stage of the analysis.

### Variant Identification by Agarose Gel Electrophoresis

**Figure 2:**
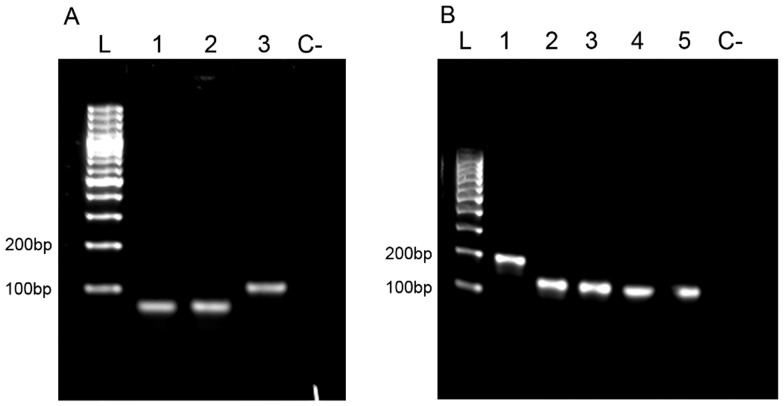
Amplicons from segments 5 and 6 resolved by agarose gel DNA electrophoresis. **A. Segment 5**. L: 100 bp ladder, 1: GF-797 (No insert Q_266_), 2: GF-552 (No insert L_266,_ 3: GF-025 (Insert IN4); C-: non template control. **B. Segment 6.** L: 100 bp ladder, 1: GF-797 (HPR0_2); 2: GF-552 (HPR2); 3: GF-041 (HPR5); 4: GF-025 (HPR7b); 5:GF-918 (HPR8); C-: non template control.

Standard DNA electrophoresis analysis is able to resolve variants in both analyzed segments (Figure 2). For segment 5, resolution was achieved for variants both containing and not containing insert IN4. Notwithstanding, this classical technique was unable to differentiate variants without insertions which also contain amino acid change Q_266_→L_266_, which corresponds to a nucleotide change A→T in the corresponding amplicon (Figure 2A, lanes 1–2). For segment 6, it was only possible to differentiate HPR0 (the largest one) from all other deleted variants (HPR2, HPR5, HPR7b and HPR8), consistently with the small-size differences in the corresponding amplicons (Figure 2B; lanes 2–5).

Since segment 5 has been reported to contain at least four different inserts although from different origins with similar sizes, in the eventuality that any of these should appear in Chile, standard DNA agarose gel electrophoresis would be unable to resolve them. In the case of segment 6, due to the existence of a large number of virulent variants primarily relying on tiny deletions, the described procedure would most likely also be limited in its ability to resolve potential new variants, therefore resulting in erroneous interpretation.

### Evaluation of the Melting Behavior of Target Samples

#### A. Under traditional melting curve measurement

**Figure 3:**
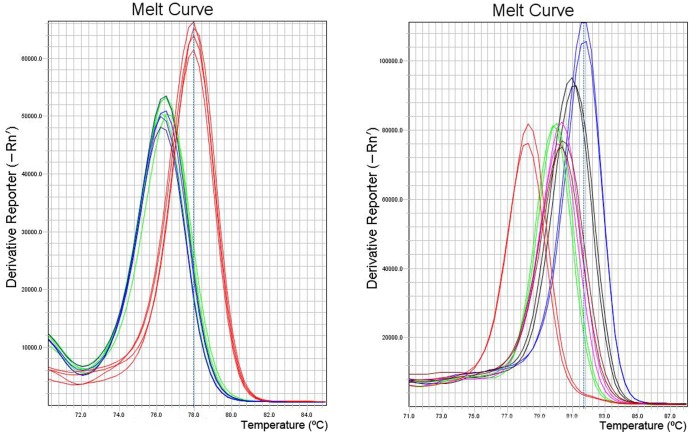
Comparative traditional melting curve analyses. A: Variants of segment 5. Blue line: GF-797 (No insert Q_266_), Green Line: GF-552 (No insert L_266_), Red Line: GF-025 (Insert IN4). B: Variants of segment 6, Blue Line: GF-797 (HPR0_2); Black Line: GF-603 (HPR0_1); Purple Line: GF-552 (HPR2); Green Line: GF-041 (HPR5); Red Line: GF-025 (HPR7b); Brown Line: GF-918 (HPR8).

Figure 3 shows the results of our evaluation of nucleotide changes in the different variants under assessment using traditional melting peak analyses. For segment 5, and as expected, it was possible to differentiate insertion-containing sequences from non-containing. Nonetheless, it was not possible to resolve non-insertion variants displaying either the A or T nucleotide change that produces the amino acid change Q_266_→L_266_, which in turn is of putative importance in virulence acquisition (Figure 3A). For segment 6, it was possible to improve discrimination between different variants as compared with the limitations encountered while using agarose electrophoresis (Figure 2B). Among all variants tested we were able to clearly differentiate the HPR7b variant from the rest; while the clustered variants HPR2, HPR5 and HPR8 were found in a very narrow area of Tm value, which meant it was not possible to resolve them. With HPR0 variants we were able to differentiate between two single nucleotide changes displaying two melting peaks: one (HPR0_1) closer to the cluster HPR2, HPR8 and HPR5 with a low melting peak temperature, and another, HPR0_2, with a distinguishable higher melting peak temperature value (Figure 3B). Recall that differentiation between HPR7b and the others variants, with the exception of the HPR0s, was not possible using the agarose gel system (Figure 2B). In summary, traditional melting peak analysis is limited in its ability to fully resolve most of the HPR variants analyzed. Table 2 summarizes the corresponding experimental peak temperature values obtained for each variant and although some differentiation is possible, the range observed is too narrow to sustain reproducible results.

**Table 2:** Experimental Melting Temperature for amplicons in segment 5 and 6.

Segment	Melting peak Temperature °C					
**5**	Insert IN4		No Insert Q_266_		No insert L_266_	
	77.94±0.07		76.45±0.14		76.35±0.00	
**6**	HPR0_1	HPR0_2	HPR2	HPR5	HPR7b	HPR8
	81.69±0.0	81.02±0.11	80.28±0.10	79.95±0.0	78.20±0.10	80.43±0.10

#### B. Under High Resolution Melting Analysis

In order to further resolve the minute differences observed in the ISAv variants analyzed in this work, we considered using HRM as an alternative and more efficient technique that despite sharing the same theoretical basis as that described for traditional melting curve analysis also presents distinguishing features that should allow enhanced resolution potential for almost identical sequences [36], [50]. HRM uses recent thermocycler technologies, especially the temperature uniformity between samples, the high rate of fluorescent data acquisition and highly sophisticated software for normalization and analysis of the melting curves. In addition, melting curve analysis is preferred as it uses novel saturating dyes that display minimal redistribution during the melting process and because, under these conditions, PCR amplification is not inhibited [34], [35], [38], [49].

**Figure 4:**
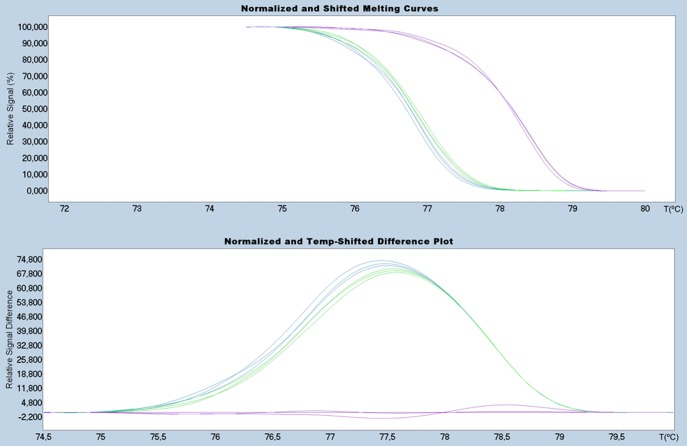
HRM analysis of segment 5: Upper Panel: Normalized melting curves; Bottom Panel: Differential plot. Red lines: GF-025 (Insert IN4) as baseline. Blue lines: GF-797 (No insert Q_266_) and Green lines: GF-552 (No insert L_266_).

We have achieved better resolution of ISAv variants for segment 5 and 6 using the HRM technique. Figure 4 shows the HRM analysis of segment 5 which differentiates between variants with and without the IN4 insertion (Table 1). A much wider resolution range is observed compared to that obtained using the traditional melting analysis (Figure 3). This is an important point for diagnosis in the event that new insertional sequences were to be detected in Chilean variants, and/or the appearance of those already describe elsewhere.

**Figure 5:**
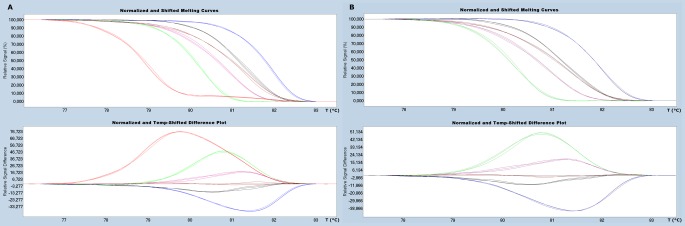
HRM analysis of segment 6: Upper Panel: Normalized melting curves; Bottom Panel: Differential plot. A: Analyses of the all variants available. B: Analysis without considering the sample GF-025. Blue Line: GF-797 (HPR0_2); Black Line: GF-603 (HPR0_1); Purple Line: GF-552 (HPR2); Green Line: GF-041 (HPR5); Red Line: GF-025 (HPR7b); Brown Line: GF-918 (HPR8) used as baseline.

Figure 5 shows the corresponding analysis for all segment 6 variants under analysis; it displays highly heterogeneous melting behavior (Figure 5A). To accurately differentiate between variants and to improve resolution and visualization of the melting curves, the following strategy was designed: first, to withdraw variant HPR7b from the analysis due to its distinguishing and different Tm value and to cluster variants HPR2, HPR5 and HPR8 plus the HPR0s, by defining different discriminatory normalization parameters (Figure 5B).

**Figure 6:**
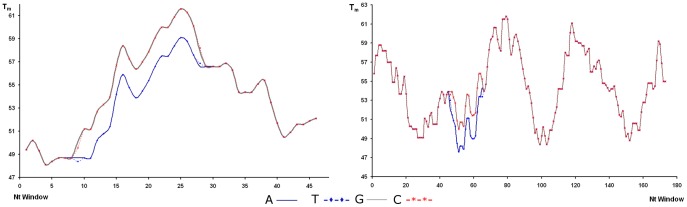
Theoretical melting profiles. **A.** Segment 5. **B.** Segment 6. Melting profiles are superimposed except for the region influenced by the nucleotide change.

As in segment 5, HRM provides a higher degree of sensitivity in resolving the wide range of variability exhibited in viral segment 6. In the previous methods discussed, it was not possible to resolve the clustered variants (Figure 3B) while HRM was able to (Figure 5). It is important to mention that under HRM analysis the melting curve profiles obtained are not related to the size of the amplicons, but instead other parameters are more relevant such as GC content and nucleotide distribution, among others. It is understandable then that although variants HPR8 and HPR7B, which are different in size by three nucleotides (one amino acid) (Table 1), exhibit completely different HRM profiles (Figure 5). HPR8 and HPR0_1 variants share similar melting curve profiles, in spite of the fact that the latter is 72 nucleotides larger than the former. Nevertheless, two samples from the HPR0 group with a single nucleotide difference upstream of the HPR region (Figure 1B) display a difference in behavior under HRM analysis (Figure 5). A similar case to the single nucleotide change for identical size amplicons observed for segment 5 could not be resolved by HRM. In the first case, the change, which involved a transversion A→C, did affect the melting profile; while in the second case the similar transversion A→T did not. In order to better understand this situation, we calculated theoretical melting temperature profiles using an algorithm of EMBOSS [51] over the amplicon considering each of the four possible nucleotides in the target site. These results are summarized in Figure 6. In either case, a change A or T ↔ C or G, modifies the profile, while changes A ↔ T or, C ↔ G, do not; this is to be expected from the thermodynamics involved in nucleotide pairing. We conclude that our theoretical analysis is coherent with our observed experimental data, suggesting that this dual analysis is projectable to other putative variants of the ISA virus. We have applied the same theoretical approach to the existing non-Chilean additional insertions reported for segment 5 (IN1– IN3), which in spite of presenting almost the same size, yielded different melting profiles (data not shown).

After optimization of the HRM analysis with reference samples, we analyzed about 20 field samples from different farm centers and sampling dates.

**Figure 7:**
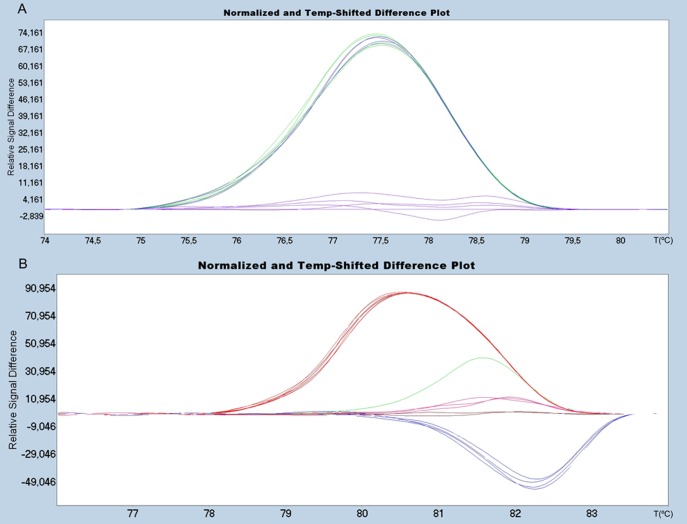
HRM analysis of field samples. **A.**Diferential plot of segment 5 insertion region: Red Line (IN4 insertion) as baseline, Blue Line (No insert Q_266_) and Green lines: GF-552 (No insert L_266_). **B.** Diferential plot of segment 6 deletion regions: Blue Line: GF-797 (HPR0_2); Purple Line: GF-552 (HPR2); Green Line: GF-041 (HPR5); Red Line: GF-025 (HPR7b); Brown Line: GF-918 (HPR8) used as baseline.

The initial processing of field samples was carried out together with the reference samples, to ensure uniform conditions and avoid bias in the analysis. Whereby, every HRM has to be analyzed independently, therefore standardization parameters must be modified according to the curves obtained in each test. Figure 7 shows the HRM curves of field samples for segment 5 and segment 6. All the variants included were resolved in an optimal way, in the same way as the reference samples.

In this report we have standardized the HRM technique specifically to fine-tune resolution between ISA virus variations exclusive to two of the most important segments associated with viral pathogenesis. It was possible to distinguish variants that have a difference in the melting temperature of only 0.15°C, as was the case with variants HPR2 and HPR8 (Table 2), which could not be separated by agarose gel or by traditional melting curve analysis.

The relevancy of the described technique should enable us to face two situations that might involve the highly variable ISAv: the coexistence of two variants in the same fish from field samples, which at present cannot be easily assessed; the appearance of new variants that might threaten the sustainability of the sector. We foresee that widening the application of the technique could be applicable to identifying variability in other segments of the ISA virus, allowing the construction of a highly necessary genetic profile of this aggressive virus. Finally, HRM allows evaluation of large numbers of samples as a presequencing screening technique. Although it does not replace DNA sequencing as a gold standard technique in differentiation between variants, HRM stands as an amicable and useful technique for determining which of many samples are recommendable for sequencing.

## Materials and Methods

### Fish Samples

The analyzed variants are organ pools (kidney, heart and gills) from naturally infected *Salmo salar* specimens, which were confirmed as positive by qRT-PCR [33] in our laboratory. Samples were collected at different times as well as from different locations from Southern Chile.

### ISA Virus Variants

The selected regions from segments 5 and 6 were sequenced (Macrogene Inc. Korea) and compared by Blast against sequences available in the GenBank database (Table 3).

**Table 3:** Variants for each sample and their corresponding access number.

Samples	Segment 5 Variant	GenBank Accession numbers of Segment 5	Segment 6 Variant	GenBank Accession numbers of Segment 6
**GF-025**	Insert IN4	EU130923	HPR7b	FJ594319
**GF-552**	No insert L_266_	GU830907	HPR2	AF364889
**GF-918**	No insert L_266_	GU830907	HPR8	AY973192
**GF-041**	Not sequenced	––––––	HPR5	EU625673
**GF-603‡**	No insert Q_266_	EU851044	HPR0_1	EU118820
**GF-797**	No insert Q_266_	EU851044	HPR0_2	EU118820

**Table 4:** Normalization parameters used in HRM analysis.

Segment	Variants used in the analyses	Pre-Melt Slider Settings	Post-Melt Slider Settings
5	All	74.5°C-75°C	79.5°C-80°C
6	All	76°C-76.5°C	83°C-83.5°C
6	Without HPR7b	77.2°C-77.6°C	83°C-83.5°C

### RNA Extraction

The organ pool samples were preserved in RNAlater (Ambion, USA) and extracted for RNA using the column-based purification method RNeasy Mini Kits (Qiagen, USA) in accordance with the standard protocols recommended by the supplier. Samples were then stored at −80°C.

### cDNA Synthesis

cDNAs were obtained through the SuperScript III reverse transcriptase (Invitrogen, USA) using random hexamer as primer (Fermentas, USA) with 1 µg of RNA in accordance with manufacturer’s instructions. Reverse transcription was performed initially with a 10 min incubation at 25°C, followed by 1 h incubation at 50°C and enzyme inactivation at 72°C for 15 min.

### Primer Design

The RNA sequences of the ISAV variants for segments 5 and 6 were obtained from GenBank database [48] using the BioEdit alignment editor [52] to find highly conserved regions in order to then design specific primers that were able to produce short amplicons. Primer 3 software [53] was used and some manual adjustments made when required, and primer properties were calculated with OligoCalc [54] which were designed outside the analyzed region for segment 5 and segment 6. For segment 5, GIM SEG-5 Ext-F (5′ TAC AAC GGA AAG GAT TAA GAC TG 3′) and GIM SEG-5 Ext-R (5′ TCT CCT TCT AGC AGC AGG TTC 3′). For Segment 6, GIM SEG-6 4F (5′ GCC CAG ACA TTG ACT GGA GTA G 3′) and GIM SEG-6 1R (5′ CTC TAG ACT TGT ACA TGA ATG CTG 3′).

### Primary PCR

2 µL of every cDNA was added to the reaction mixture with a final volume of 12.5 µL containing 2 mM MgCl_2_, 1X Go Taq Buffer, 0.5 U Go taq enzyme (Promega, USA), 0.2 mM each dNTPs (Promega, USA), and 0.5 µM of each primer. The cycling conditions were 1 cycle of the initial denaturation at 95°C for 2 min, then 35 cycles at 95°C for 30 s, followed by primer annealing at 60°C for 30 s and an extension step at 72°C for 1 min. A final extension cycle was obtained for 5 min at 72°C.

The product of this amplification was diluted 1000 times to be used in the amplification through real-time PCR and the High Resolution Melting curve analyses were then performed.

### Agarose Electrophoresis

Every PCR-amplified sample was resolved by 2% (w/v) agarose gel electrophoresis in a horizontal eletrophoresis chamber at 100 V for 30 min and visualized in GelRed stained gels (PhotoCapture; DNR Bio-imaging System, Ltd. Israel).

### Traditional Melting Curve Analyses

This analysis was performing in a Real time thermocycler Step OnePlus (Applied Biosystems. USA). The procedure was carried out using the same protocols, reagents and temperature program used for the secondary real-time PCR and HRM analysis (detailed in the next section). The traditional melting curve measurements were performed using fluorescent data acquisition every 0.3°C. The analysis was carried out by derivative melting curve plot. The melting peak temperature determination was performed with the Applied Biosystem StepOne v2.2 software.

### Secondary Real-Time PCR and HRM Curve Analysis

Real–time PCR amplification and acquisiton of the HRM curve was carried out with the thermocycler LightCycler 2.0 (Roche Diagnostics, Germany). The reagent mix was prepared with 2 µL of the diluted primary PCR, 2.5 mM MgCl_2_, 1X LightCycler 480 HRM master mix (Roche, Germany), 200 nM of each primer, in a final reaction volumen of 20 µL. PCR cycles were performed under the following conditions: 1 cycle at 95°C for 10 min; 40 cycles at 95°C for 10 s, 60°C for 10 s, and 72°C for 20 s. The amplicons were melted by raising the temperature from 70°C to 90°C, with an increment of 0.05°C/s and fluorescence was continuously acquired, in order to obtain information on melting profiles.

The analysis of the HRM curve was performed using the LightCycler 480 gene-scanning Software v1.5. This software analyzes the HRM curve data to identify the shape of the curve and the Tm differences. The software employs a three-step analysis. The first step is to normalize the raw melting-curve data by setting the pre-melt (initial fluorescence) and post-melt (final fluorescence) signals of all samples to uniform values. Pre-melt signals are uniformly set to a relative value of 100%, while post-melt signals are set to a relative value of 0%. The normalization parameters used are summarized in Table 4. The second step is to shift the temperature along the X-axis of the normalized melting curves at the point where the entire double stranded DNA is completely denatured. The third step is to further analyze the differences in melting-curve shape by subtracting the curves from a reference curve (also called the “baseline”). Thus generating a difference plot, accentuating the changes in the shapes of the melting curves [43].

Due to the diversity of the samples, the first parameter to discriminate between the variants was the Tm value.

We carried out the first and third step of the software analysis independently. The second step, which unifies the final melting temperature, is used mainly in HRM applications to identify heterozygosity [49]. In the present study, the presence of insertions or deletions in the samples was sufficient to produce a temperature change. Nevertheless, if it is necessary to differentiate minimal changes between two sequences with the same Tm it will be necessary to perform this step to improve resolution.

### Theoretical Calculation of Melting Temperature Profiles

For theoretical melting profiles we used the application Dan of EMBOSS suite [51], through Mobyle portal [55]. The melting profile is calculated as the average within a sliding window taking into account the thermodynamic behavior of the nearest neighbor [56], [57]. We chose a 20 nucleotide window as reference. For both segments, 5 and 6, the target position was replaced by each of the four nucleotides (ATGC), and the resulting profiles are shown in Figure 6.
